# U94 of human herpesvirus 6 down-modulates Src, promotes a partial mesenchymal-to-epithelial transition and inhibits tumor cell growth, invasion and metastasis

**DOI:** 10.18632/oncotarget.17817

**Published:** 2017-05-11

**Authors:** Francesca Caccuri, Roberto Ronca, Andrea S. Laimbacher, Angiola Berenzi, Nathalie Steimberg, Federica Campilongo, Pietro Mazzuca, Arianna Giacomini, Giovanna Mazzoleni, Anna Benetti, Elisabetta Caselli, Marco Presta, Dario Di Luca, Cornel Fraefel, Arnaldo Caruso

**Affiliations:** ^1^ Department of Molecular and Translational Medicine, University of Brescia, Brescia, Italy; ^2^ Institute of Virology, University of Zurich, Zurich, Switzerland; ^3^ Department of Clinical and Experimental Sciences, University of Brescia, Brescia, Italy; ^4^ Department of Medical Sciences, University of Ferrara, Ferrara, Italy

**Keywords:** HHV-6 U94, HSV-1 amplicon vector, mesenchymal-to-epithelial transition, Src signaling pathway, cancer development and metastasis

## Abstract

U94, the latency gene of human herpesvirus 6, was found to inhibit migration, invasion and proliferation of vascular endothelial cells (ECs). Because of its potent anti-migratory activity on ECs, we tested the capability of U94 to interfere with the individual steps of the metastatic cascade. We examined the U94 biological activity on the human breast cancer cell line MDA-MB 231, as a model of highly aggressive cancer cell. Here we show that the expression of U94 delivered by an HSV-1-based amplicon promoted down-modulation of *Src* and downstream molecules linked to cell motility and proliferation. Indeed, U94 expression strongly inhibited cell migration, invasiveness and clonogenicity. We investigated the effects of U94 in a three-dimensional rotary cell-culture system and observed the ability of U94 to modify tumor cell morphology by inducing a partial mesenchymal-to-epithelial transition. In fact, despite U94 did not induce any expression of the epithelial marker E-cadherin, it down-modulated different mesenchymal markers as β-catenin, Vimentin, TWIST, Snail1, and MMP2. *In vivo* data on the tumorigenicity of MDA-MB 231 displayed the capability of U94 to control tumor growth, invasiveness and metastasis, as well as tumor-driven angiogenesis. The antitumor U94 activity was also confirmed on the human cervical cancer cell line HeLa. The ability of U94 to inhibit cell growth, invasion and metastasis opens the way to a promising field of research aimed to develop new therapeutic approaches for treating tumor and cancer metastasis.

## INTRODUCTION

Latency is an evolutionary mechanism that some viruses have exploited in order to survive in a hostile microenvironment. Chief among virus families known to be capable of latency are the herpesviruses, a widely distributed family of large, enveloped DNA viruses that are important pathogens in the human host. During herpesvirus latency, only a handful of genes are expressed, being able to silence most viral genes linked to active virus replication and, in turn, to modulate different host cell functions, particularly those affecting the life span and proliferative potential of the latently infected cells. The human beta-herpesvirus subfamily includes two viruses, herpesvirus type 6 (HHV-6) A and B. These two viruses share molecular mechanisms of viral latency, during which specific transcripts from the viral gene U94 are expressed [1]. Interestingly, U94 displays DNA binding [2], exonuclease and helicase-ATPase activities [3]. In addition, U94 inhibits gene transcription and cell transformation by Harvey (H)-*ras* and bovine papillomavirus type 1 (BPV-1) viruses [4] as well as transcription from the human immunodeficiency virus type 1 (HIV-1) and human papillomavirus type 16 (HPV-16) [5]. Such activities suggest a role for U94 in viral gene regulation and DNA replication.

More recently, human endothelial cells (ECs) were found to be susceptible to HHV-6 infection [6, 7] forming a site where the virus can persist in the absence of cytopathic effect and establish a latent infection. U94 expression in ECs in the absence of other viral transcripts was found to be associated to inhibition of different angiogenetic steps. In particular, U94 expression strongly inhibited *in vitro* capillary-like structures formation, sealing of a mechanical injured EC monolayer, angiogenesis and vasculogenesis *ex vivo* [8], all activities linked to the control of migration, invasion and proliferation of vascular ECs.

In this report, we explore the *in vitro* U94 activity on two different human cancer cell lines and provide evidence that the viral protein down-modulates the proto-oncogene *Src* activation and downstream signaling pathways. At the same time, we found that U94 expression induces a partial mesenchymal-to-epithelial transition and impairs cell migration, invasion and proliferation. Data on the tumorigenicity in NOD/SCID mice showed that despite a rapid loss of the U94 transgene expression, the viral protein does exert a long-term control of tumor growth, invasiveness and metastasis.

## RESULTS

### U94 expression in amplicon-transduced cells

**Figure 1:**
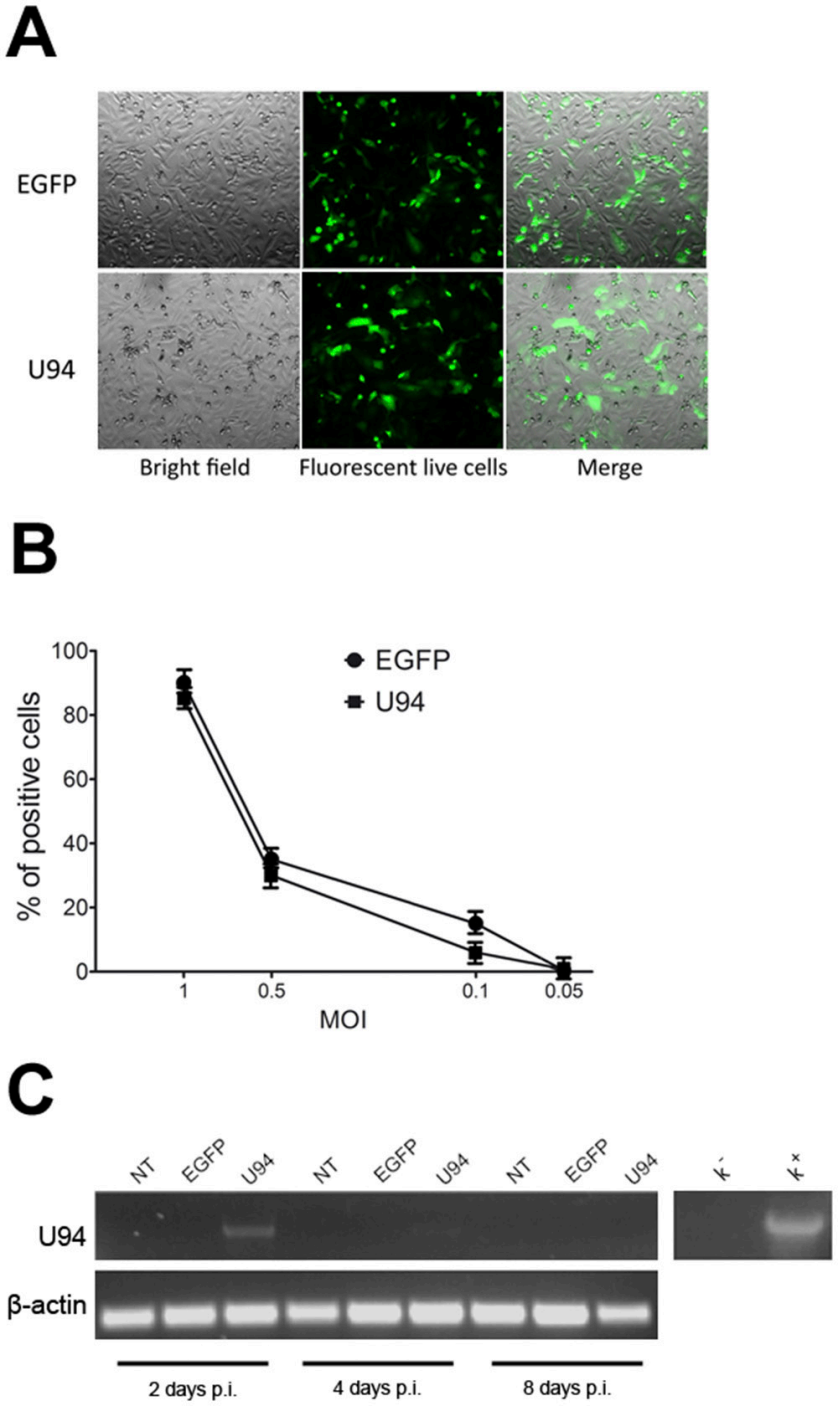
HSV-1 amplicons titration and characterization **(A)** HSV-1 amplicon constructs were transduced into Vero 2-2 cells and EGFP expression was visualized by fluorescence microscopy. One day after infection, single cells expressing EGFP were representative of gene expression and cell transduction. In the right panel fluorescence images merged with corresponding bright field images to show Vero 2-2 cell morphology (original magnification 10x). **(B)** MDA-MB 231 cells were infected with amplicon vectors at different MOI and the EGFP expression was evaluated by flow cytometry. The percentage of positive cells is reported in the graph. **(C)** The presence of U94 mRNA was analyzed by RT-PCR in MDA-MB 231 cells infected with amplicon constructs at different days p.i. K^−^, negative control, water; K^+^, positive control, plasmid expressing U94.

Amplicons were titrated on Vero 2-2 cells (Figure 1A). To define the optimal condition to obtain a maximum number of U94-expressing (U94^+^) cells, MDA-MB 231 cells were infected at different MOI and EGFP fluorescence was measured by flow cytometry. The highest efficiency of viral infection (range from 80 to 93%) was obtained at MOI 1 for all tested constructs (Figure 1B). The persistence of U94 expression in MDA-MB 231 cells was verified by RT-PCR analysis (Figure 1C). U94 transcripts were detected at day 2 post infection (p.i.), whereas a faint or no expression was evident at day 4 and 8 p.i., respectively (Figure 1C).

### U94 inhibits cell proliferation

**Figure 2:**
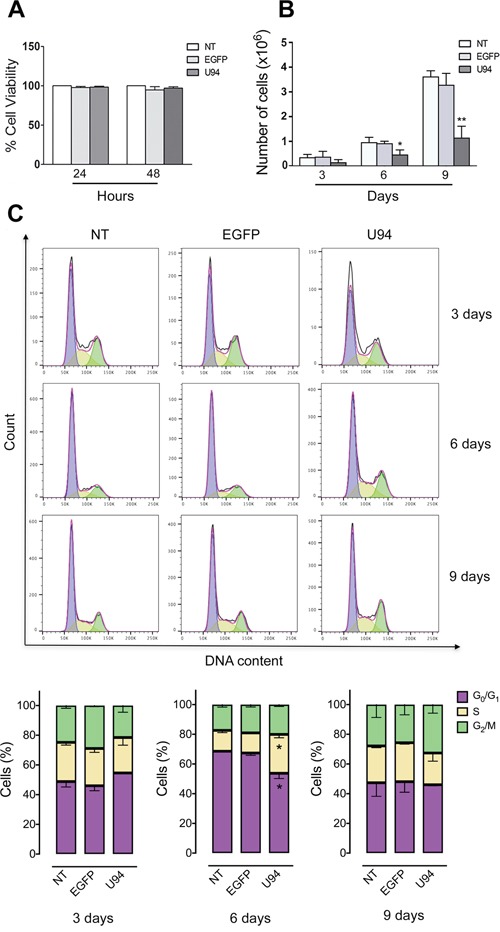
U94 expression inhibits cell proliferation and transiently arrests cells in S-phase **(A)** The number of viable cells in culture was determined by quantification of the ATP, which signals the presence of metabolically active cells. Data are shown as cell viability (%) by comparing the infected groups with the NT cells, the viability of which was assumed to be 100%. **(B)** At each time point, cells were counted using the trypan blue exclusion method. Bars represent the mean ± SD of three independent experiments performed in triplicate. **(C)** On the days indicated, U94^+^, EGFP^+^ and NT cells were stained with propidium iodide and the cell cycle profile was determined by flow cytometry. Upper panels, FACS analysis is representative of three independent experiments with similar results. Lower panels, data (mean ± SD) are representative of three independent experiments. Statistical analysis was performed by 1-way ANOVA, and the Bonferroni post-test was used to compare data (*P < 0.05).

No toxicity was observed in MDA-MB 231 cells infected for 48 h with the different amplicon vector stocks compared to not treated (NT) cells (Figure 2A). However, at day 6 and 9 p.i., a significant reduction in cell proliferation was observed in U94^+^ cells compared to control EGFP-expressing (EGFP^+^) or NT cells (Figure 2B). We measured cell cycle distribution of U94^+^ cells and found a significant arrest in the S-phase at day 6 p.i., compared to EGFP^+^ and NT cells (Figure 2C). This arrest was transient since it was not detected at day 9 p.i. In contrast, an increased – even if not statistically significant – G_2_/M cell cycle entry of U94^+^ cells, as compared to control, cells is indicative of active cell division at day 9 p.i. This finding attests for a reversible S-phase block operated by U94, possibly related to the transient expression of the viral protein in MDA-MB 231 cells.

### U94 inhibits cancer cell motility, invasion and anchorage-independent growth

**Figure 3:**
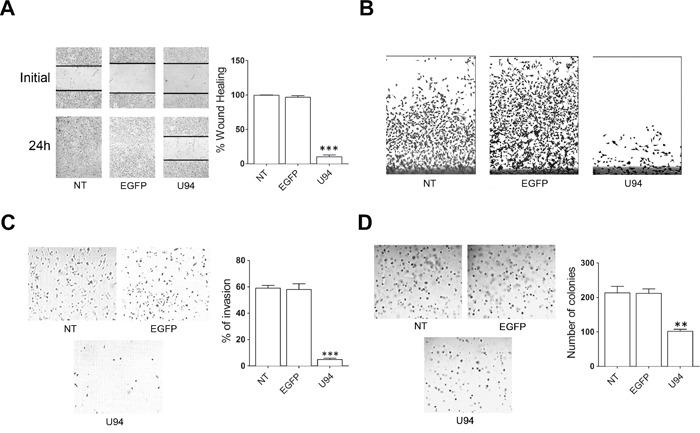
U94 expression inhibits MDA-MB 231 cell migration, invasion and clonogenicity **(A)** Confluent cell monolayers were scratched using a 200 μl pipette tip and sealing of the wound was recorded by light microscopy over a 24 h time course after wound scratch (original magnification 10x). **(B)** Cell movement along the bottom of an angled flask was recorded at day 8 p.i. **(C)** Cell invasion assay was performed by Matrigel-coated transwell system. Cells were resuspended in a serum-free medium and seeded in the upper chamber. Complete medium was used as chemoattractant factor in the lower chamber. After 48 h of culture, migrated cells were stained, photographed and counted (original magnification 10x). **(D)** Colony forming ability of MDA-MB 231 cells infected or not with amplicons. Cells were plated into six-well plates and, after two days, the medium was replaced using fresh medium. Cell growth was analyzed by using MTT (original magnification 10x). Data represent the average of three independent experiments performed in triplicate. Images are representative of three independent experiments with similar results. Statistical analysis was performed by 1-way ANOVA, and the Bonferroni post-test was used to compare data (** *P* < 0.01; *** *P* < 0.001).

The capability of U94 to interfere with the migratory activity of cells was assessed by wound healing assay. NT or EGFP^+^ MDA-MB 231 cells reached 100% of sealing 24 h after the wound. At the same time, U94^+^ cells reached 10% of sealing only (range from 5% to 15%), showing a strong inhibition in wound repair ability (Figure 3A). Cell movement along the bottom of an angled flask was recorded after 8 days. Cell migration strongly decreased in U94^+^ cells as compared to NT or EGFP^+^ cells, attesting for a long-standing anti-migratory efficacy of U94 (Figure 3B). MDA-MB 231 cells are highly aggressive and metastatic, and tumor cell invasion is one of the key steps in this complex process. As the metastatic potential of tumor cells is largely dependent on their ability to degrade and migrate through the extracellular matrix (ECM), we examined the ability of NT, U94^+^ and EGFP^+^ MDA-MB 231 cells to invade ECM. Fourthy-eight h p.i., invasion of U94^+^ cells was strongly reduced as compared to NT or EGFP^+^ cells (Figure 3C, left panel). Quantitative analysis demonstrated that only 4.9% of U94^+^ cells were able to invade the matrigel and the filter compared to 58% of EGFP^+^ and 59% of NT cells (Figure 3C, right panel). This result suggests that U94^+^ MDA-MB 231 cells have a much lower invasive potential than control cells *in vitro*. The ability for cancer cells to migrate and survive in the circulation is required for metastasis dissemination [9]. Adaptation to new environment is a hallmark of aggressive tumors and to survive, cancer cells have to grow and expand in the absence of attachments by overcoming *anoikis* [10]. The expression of U94 significantly inhibited the colony-forming ability of MDA-MB-231 cells compared to EGFP^+^ or NT cells (Figure 3D, left panel) in an anchorage-independent environment (soft agar). In fact, an approximately 60% decrease in colony number was observed in U94^+^ (91 ± 7) compared to EGFP^+^ or NT cells (222 ± 14 and 223 ± 21, respectively) (Figure 3D, right panel).

### U94 expression in MDA-MB 231 cells leads to a partial mesenchymal-to-epithelial transition (MET)

**Figure 4:**
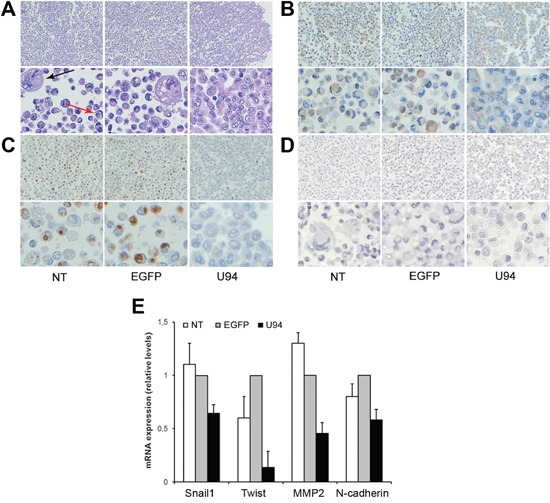
U94 expression induces a partial MET in MDA-MB 231 cells After 3D culture, cells were harvested and processed for staining. Five μm thick sections were prepared and stained with **(A)** H&E for morphological analysis, or IHC for **(B)** β-catenin, **(C)** Vimentin, and **(D)** E-cadherin expression. (Original magnification: 20x upper panels; 40x lower panels). The arrows in panel A show cytonuclear atypias, such as nuclear pleomorphism, multinuclearity (black arrow) and “cell in cell” aspects (red arrow). **(E)** Real-time-PCR analysis of mesenchymal markers Snail1, Twist, MMP-2 and N-Cadherin was performed on NT cells and on EGFP^+^ and U94^+^ cells at day 4 p.i. Gene expression is represented as relative mRNA levels. Data represent the mean (± SD) of three independent experiments performed in triplicate.

In order to study cell-cell interaction upon U94 expression, we used the Rotary Cell Culture System (RCCSTM, Synthecon, Inc.) to generate a 3D cell culture microenvironment. After two days in 3D fluid-dynamic culture, spheroids formed by NT, U94^+^ or EGFP^+^ MDA-MB 231 cells were harvested as previously described [11] and morphologically examined by the classical hematoxylin and eosin (H&E) staining. As shown in Figure 4A, more than 30% of NT and EGFP^+^ cells appeared isolated and characterized by nuclear and cytoplasmic alteration as pleomorphism, multinuclearity and “cell in cell” aspect. On the other hand, U94 expression was related to disappearance of atypias and increased cell-cell adhesion. Based on these data, we sought to understand if U94 was able to trigger a MET. In normal epithelial cells, β-catenin exhibits membranous staining, whereas excess of free β-catenin in the cytoplasm is rapidly degraded [12]. In transformed cells, excess of β-catenin accumulates in the cytoplasm owing to impairment of its degradation [13], leading to its translocation into the nucleus, where it acts as transcriptional factor able to activate several genes involved in oncogenesis [14, 15]. Immunohistochemistry (IHC) of spheroids showed a cytoplasmic expression of β-catenin in control cultures, whereas it was mostly (≥ 90% of cells) confined at the cell membrane in U94^+^ cultures, (Figure 4B). Since relocation of β-catenin from cytoplasm to cell membrane is indicative of a switch of tumor cells from an aggressive to a more differentiated phenotype, our data corroborate the hypothesis on the role of U94 in triggering a MET. Vimentin is another marker of epithelial-to-mesenchimal transition (EMT), whose expression is transactivated by β-catenin [16]. Vimentin is overexpressed in several aggressive breast cancer cell lines and correlated with increased tumor cell migration and invasion [17]. Loss of vimentin expression exhibits inhibition of motility and invasiveness of MDA-MB 231 cells, which constitutively express vimentin [18]. As shown in Figure 4C, vimentin was found to be expressed as cytoplasmic dots in less than 2% of U94^+^ cells, whereas approximately 45% of control cells did express it as both diffuse staining and punctate dots in the cytoplasm. On the other hand, any attempt to detect E-cadherin, an epithelial marker and suppressor of tumor cell invasion and metastasis in U94^+^ MDA-MB 231 cells was unsuccessful (Figure 4D). As demonstrated by real-time-PCR, U94 expression also induced a strong down-modulation of TWIST, a well-established transcription factor inducing EMT and promoting tumor invasion and metastasis [19], as well as a concomitant down-regulation of the mesenchymal markers N-cadherin, Snail1, and MMP2 (Figure 4E). Taken together these results support a role for U94 in triggering a partial MET of MDA-MB 231 cells.

### U94 inhibits *Src* and downstream signaling cascade

**Figure 5:**
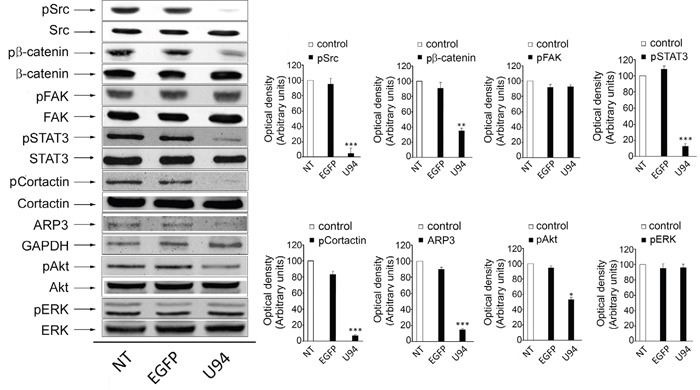
Effect of U94 on cell signaling pathways Western blot analysis of MDA-MB 231 lysates was performed using mAbs to Src pTyr418, total Src, β-catenin pTyr654, total β-catenin, FAK pTyr397, total FAK, STAT3 pTyr705, total STAT3, Cortactin pTyr421, total Cortactin, ARP2/3, GAPDH, Akt pSer473, total Akt, ERK pThr202 and total ERK as specific reagents. Quantification was carried out by densitometric analysis and plotting of the Src pTyr418/Src, β-catenin pTyr654/β-catenin, FAK pTyr397/FAK, STAT3 pTyr705/STAT3, Cortactin pTyr421/Cortactin, ARP2/3/GAPDH, Akt pSer473/Akt and ERK pThr202/ERK. Left panels, blots are representative of three independent experiments with similar results. Right panels, values reported are the means ± the SD of three independent experiments. Statistical analysis was performed by 1-way ANOVA, and the Bonferroni post-test was used to compare data (* *P* < 0.05; ** *P* < 0.01; *** *P* < 0.001).

Western blot analyses were performed to determine whether U94 effects on MDA-MB 231 cells were mediated by down-modulation of signaling pathways usually involving tumor cell motility, invasion and proliferation. As shown in Figure 5, immunoblotting of U94^+^ MDA-MB 231 cell lysates showed that p*Src* (pY418) levels dramatically decreased as compared to control cells (Figure 5, left panels). Down-modulation of p*Src* resulted in the concomitant inactivation of β-catenin (pY654), STAT3 (pY705), Cortactin (pY421), ARP2/3 and Akt (pS473). Quantification of data from multiple experiments are shown in Figure 5 (right panels). At the same time, EGFP^+^ cells did not show any inhibition of the above signaling pathways when compared to NT cells. Specificity of inhibition of the proto-oncogene *Src* and its downstream substrates by U94 was confirmed by the evidence that other signaling pathways summoned to promote cancer cell invasion and metastasis, such as FAK and ERK [20], were not affected by the viral protein (Figure 5).

### U94 expression impairs tumor growth in NOD/SCID mice

**Figure 6:**
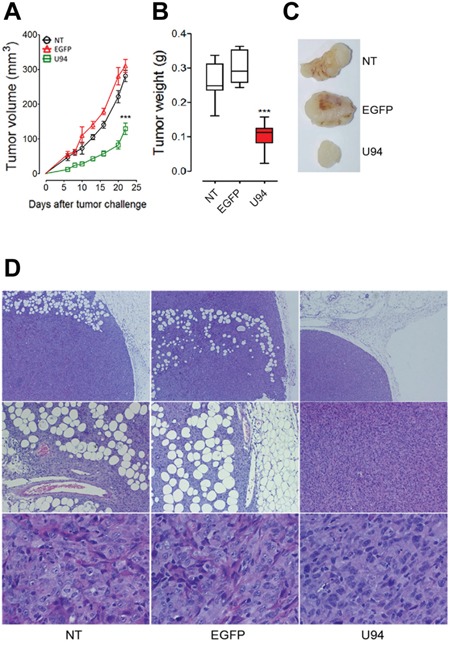
U94 expression impairs tumor growth *in vivo* Twenty-tree days after cell injection, mice were sacrificed; tumors were **(A)** measured and **(B)** weighted. **(C)** a representative picture of one tumor of each group is reported (n=8-10 mice/group). Data are mean ± SEM. In box and whiskers graphs, boxes extend from the 25^th^ to the 75^th^ percentiles, lines indicate the median values, and whiskers indicate the range of values. (*** *P* < 0.001). Tumors were processed for IHC. Five μm thick sections were prepared and stained with **(D)** H&E for morphological analysis. (Original magnification: 10x upper panels; 20x middle panels 40x lower panels).

**Figure 7:**
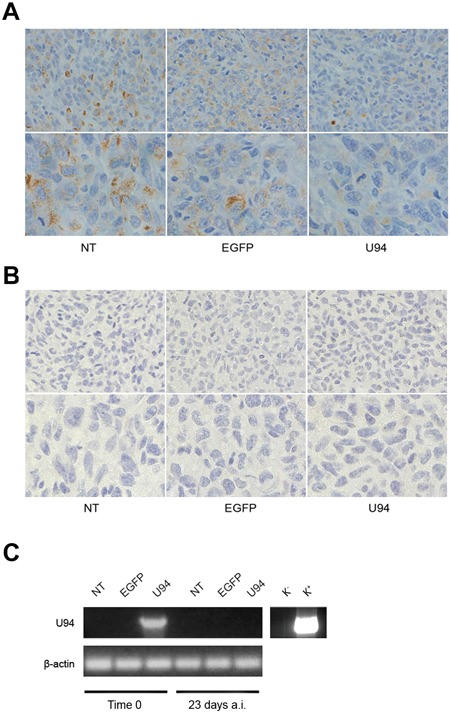
U94 induces MET of xenografted tumor cells Five μm thick sections were prepared and stained with antibodies against **(A)** vimentin and **(B)** E-cadherin (Original magnification: 20x upper panels; 40x lower panels). **(C)** the presence of U94 mRNA was analyzed by RT-PCR in MDA-MB 231 cells infected with HSV-1 amplicon constructs before injection in mice (Time 0) and in tumor biopsies 23 days after injection (a.i.). K^−^, negative control, water; K^+^, positive control, plasmid expressing U94.

To further investigate the effect of U94 on tumor progression *in vivo*, NT, EGFP^+^ or U94^+^ MDA-MB 231 cells were injected into the dorsolateral flank of NOD/SCID mice. Tumor growth was monitored and tumors were excised and weighed 23 days after injection. U94 expression caused a dramatic delay of tumor take when compared to NT or EGFP^+^ tumors. U94^+^ tumors resulted to be smaller compared to control tumors, suggesting that U94 supports inhibition of tumor growth (Figure 6A). The average weight of tumors derived from NT, EGFP^+^ and U94^+^ cells were 0.26 ± 0.02, 0.29 ± 0.01 and 0.10 ± 0.01 g, respectively (Figure 6B). At day 23 after tumor cells injection, tumor growth was strongly inhibited in each mouse inoculated with U94^+^ cells compared to controls (Figure 6C). IHC analysis of biopsies revealed consistent morphological differences between tumors generated by U94^+^ cells and control tumors generated by NT or EGFP^+^ cells. H&E staining of U94^+^ xenografted tumors showed that nodules were small, closely knit and very well defined. On the contrary, control tumor biopsies were characterized by fat invasion, which is a poor prognosis factor for cancer development [21] (Figure 6D, upper panels) and distinguished by the presence of blood vessels which almost missed in U94^+^ tumors (Figure 6D, middle panels). Moreover, control biopsies presented severe cellular atypias attested by the presence of clearly visible spindle-shaped cells as compared to U94^+^ biopsies (Figure 6D, lower panels). Moreover, IHC analysis of biopsies showed that U94^+^ xenografted tumors displayed weak dot-like cytoplasmic expression of vimentin, whereas tumors generated by NT or EGFP^+^ cells showed abundant and largely diffused cytoplasmic expression of vimentin (Figure 7A). As expected, the expression of E-cadherin was not detected in NT and EGFP^+^ as well as in U94^+^ biopsies (Figure 7B). *In vivo* results confirm the ability of U94 to promote a partial MET. RT-PCR analysis of biopsies revealed that U94 transcripts were expressed in U94^+^ cells before their injection into mice but not in the excised tumors (Figure 7C). This result suggests that even if expression of U94 transcripts is limited at first stages of tumor engraftment, this is sufficient to strongly inhibit tumor growth and local invasion.

### U94^+^ tumor cells are able to condition the microenvironment inducing lack of angiogenesis in xenografted tumors

**Figure 8:**
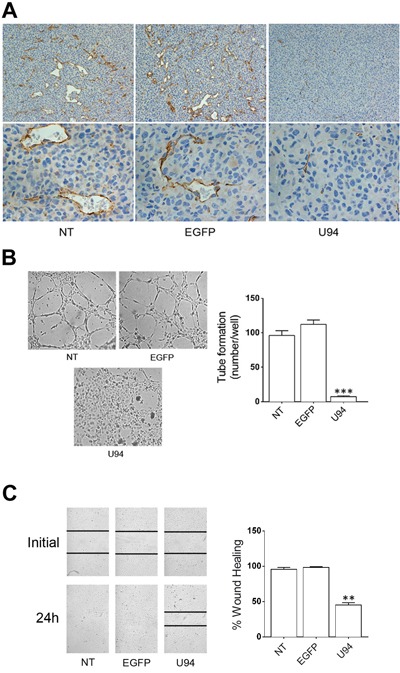
U94 inhibits tumor-driven angiogenesis **(A)** CD31 immunostaining of tumor biopsies (original magnification: 20x upper panels; 40x lower panels). **(B)** NT, EGFP^+^ and U94^+^ MDA-MB 231 cells were co-cultured with HUVECs. Twenty-four h after co-culture, HUVECs were seeded on BME-coated plates. Images were taken after 8 h of HUVEC culture on BME (original magnification 10x). **(C)** HUVEC confluent monolayers were scratched using a 200 μl pipette tip and cultured in the presence of medium obtained from NT, EGFP^+^ or U94^+^ MDA-MB 231 cells. Wound sealing was recorded by light microscopy over a 24 h time course after wound scratch (original magnification 10x). Data are representative of three independent experiments with similar results. Statistical analysis was performed by 1-way ANOVA, and the Bonferroni post-test was used to compare data (** *P* < 0.01; *** *P* < 0.001).

A polyclonal antibody to mouse CD31 was used to identify blood vessels infiltrating xenografted tumors. IHC analysis showed that tumors derived from NT or EGFP^+^ cells were highly vascularized (Figure 8A). On the other hand, sections of tumors derived from U94^+^ cells showed a decreased number of tiny blood vessels with a small – and sometime lacking – lumen. The evidence of a strongly impaired vasculogenesis in tumor sections derived from U94^+^ cells is quite surprising, being that the neo-vasculature is of mouse origin and does not express U94. Previous *ex vivo* data showed that U94 treatment renders rat aortic rings insensitive to the potent VEGF-induced vasculogenetic activity [8]. This finding suggests the capability of U94^+^ xenografts to promote a tumor microenvironment unfavorable to neo-vessel formation. To test this hypothesis, we investigated the effect of U94^+^ MDA-MB 231 cells conditioned culture medium on the angiogenetic activity of HUVECs. As shown in Figure 8B, HUVECs co-cultured for 24 h in the collagen-coated upper insert well of a 3 μm pore-size Transwell with U94^+^ cells in the lower chamber, completely lost their natural ability to form tube-like structures when detached and seeded on Matrigel. On the contrary, HUVECs co-cultured with NT or EGFP^+^ cells were capable of exerting angiogenesis, forming a consistent network of tube-like structures. Moreover, the presence of conditioned medium obtained from U94^+^ MDA-MB 231 on HUVECs monolayers impaired sealing of the wound, whereas this did not occur in HUVECs cultured with medium obtained from NT or EGFP^+^ MDA-MB 231 cells during the wound healing assay (Figure 8C). Even if mechanisms of inhibition remain to be elucidated, these data strongly support the hypothesis of a key role played by U94^+^ tumor cells in impairing vasculogenesis *in vivo*.

### U94 decreases the ability of cancer cells to form metastasis *in vivo*

**Figure 9:**
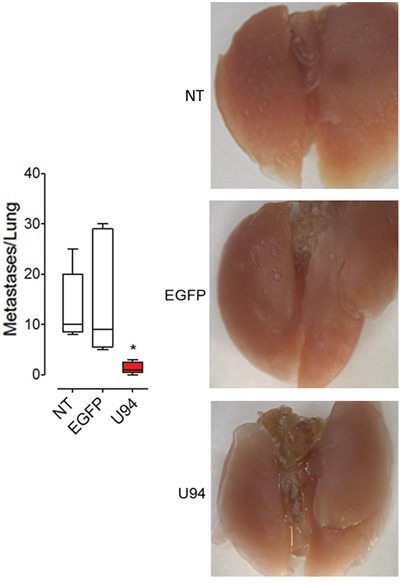
U94 strongly inhibits lung metastatic foci formation MDA-MB 231 cells were injected intravenously in mice (n=10 mice/group). After 4 weeks, lungs were collected and macroscopic metastases were counted (left panel). A representative picture of one lung of each group is reported (right panels). Data are mean ± SEM. In box and whiskers graphs, boxes extend from the 25^th^ to the 75^th^ percentiles, lines indicate the median values, and whiskers indicate the range of values. (* *P* < 0.05).

Additional *in vivo* experiments were performed to examine whether U94 is also able to modulate experimental lung metastasis formation. MDA-MB 231 cells expressing or not U94 were injected into immunocompromised mice via the tail vein. Significantly reduced numbers of lung metastasis was observed in the U94^+^ group compared with the EGFP^+^ and NT control groups (Figure 9, left panel). Representative images are shown in Figure 9, right panels. Taken together our results suggest an inhibitory role of U94 in cancer progression, including experimental lung metastasis formation.

### U94 is able to inhibit cell migration and proliferation of a different tumor cell line

**Figure 10:**
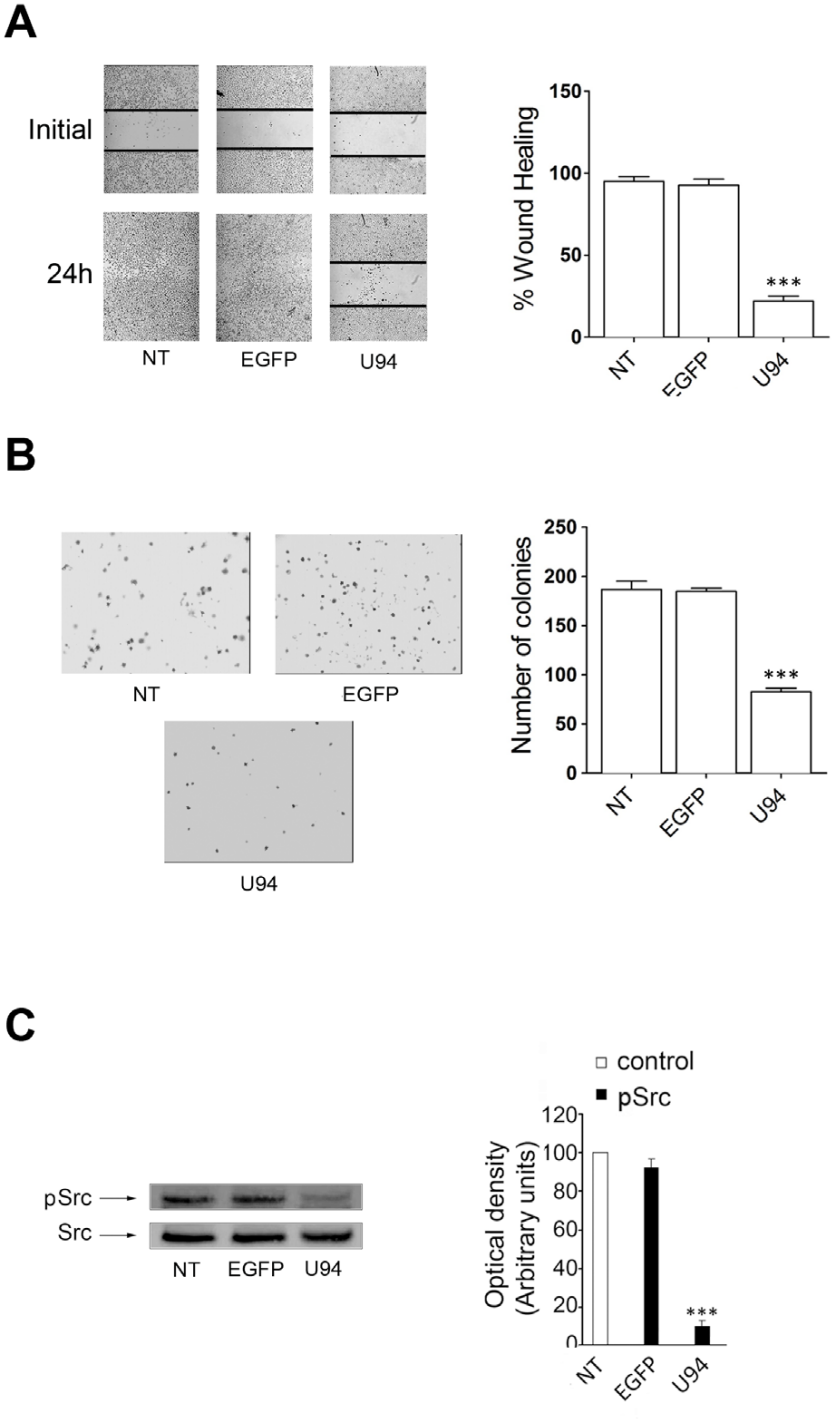
*In vitro* effect of U94 on HeLa cells **(A)** wound healing assay, **(B)** cell clonogenic assay and **(C)** signaling pathway assay. Data represent the average of three independent experiments performed in triplicate. Images are representative of three independent experiments with similar results. Statistical analysis was performed by 1-way ANOVA, and the Bonferroni post-test was used to compare data (*** *P* < 0.001).

**Figure 11:**
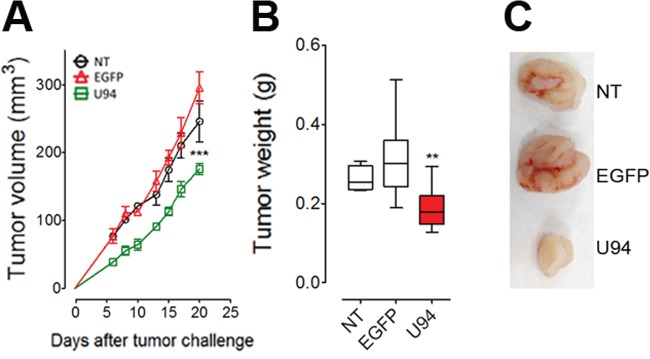
U94 expression on HeLa cells reduces xenografted tumor growth Twenty-three days after HeLa cell injection, mice were sacrificed; tumors were **(A)** measured and **(B)** weighted. **(C)**, a representative picture of one tumor of each group is reported. (n=8-10 mice/group). Data are mean ± SEM. In box and whiskers graphs, boxes extend from the 25^th^ to the 75^th^ percentiles, lines indicate the median values, and whiskers indicate the range of values. (** *P* < 0.01; *** *P* < 0.001).

The U94 inhibitory activity of migration and proliferation was also evaluated on HeLa – a human cell line derived from cervical cancer cells – by wound healing and soft-agar assays. The experiments were conducted at 24 h p.i. with EGFP- and U94-expressing plasmids. At the end of the wound healing assay, control HeLa cells reached 100% of sealing, whereas cells transduced with U94 reached 25.8% healing only (range from 20.9% to 30.6%) (Figure 10A). In the soft agar assay, expression of U94 significantly inhibited cell colony formation as compared to control cells. In particular, the colony number decreased from 189 ± 18 and 180 ± 3 for NT and EGFP^+^ cells respectively, to 90 ± 10 for U94^+^ cells (Figure 10B). Similarly to what we observed in MDA-MB 231, U94-triggered effects on HeLa cells were associated with *Src* down-modulation (Figure 10C). To further investigate the effect of U94 on human cervical cancer progression *in vivo*, NT, EGFP^+^ or U94^+^ HeLa cells were injected into the dorsolateral flank of NOD/SCID mice. As for MDA-MB 231 tumors, U94 expression caused a dramatic delay of tumor take when compared to NT or EGFP^+^ tumors. As shown in Figure 11A, tumor growth rate significantly decreased during the follow-up period, suggesting that U94 supports inhibition of HeLa tumor growth. The average weight of tumors derived from NT, EGFP^+^ and U94^+^ cells were 0.26 ± 0.01, 0.31 ± 0.03 and 0.18 ± 0.01 g, respectively (Figure 11B). At day 23 after tumor cells injection, HeLa tumor growth was strongly inhibited in each mouse inoculated with cells expressing U94 as compared to controls (Figure 11C).

## DISCUSSION

The ability of tumor cells to mobilize, invade and cross normally non-permissive tissue barriers is responsible for cancer dissemination. A necessary first step in metastasis consists in epithelial cancer cell transition to a mesenchymal-like element via EMT. EMT comprehends coordinated molecular and cellular changes leading epithelial cancer cells to express mesenchymal markers, display a reduction in cell-cell adhesion and gain motility [22, 23]. After undergoing EMT, tumor cells can extravasate into secondary organs, where cancer cells establish a new niche for tumor cell attachment and growth [24, 25]. Inhibiting or reversing an EMT can be regarded as a safe strategy for limiting cancer invasion and progression.

The *Src* family members are classified as oncogenic proteins for their involvement in all aspects of tumor development and microenvironment remodeling [26]. Increased *Src* activity drives EMT by inducing: i) cytoplasmic accumulation of β-catenin leading to dissociation of cell-cell junctions [27]; ii) β-catenin transcriptional activity that in turn promotes EMT through Snail1 over-expression; iii) tyrosine phosphorylation of cortactin leading to cytoskeleton activation and cell motility [28]; and iv) activation of Twist, that promotes the generation of an aggressive cancer stem cell phenotype [29]. On the other hand, *Src* inhibition suppresses the EMT process *in vitro* and *in vivo* [30–32].

The results of the present study reveal the capability of the HHV-6 protein U94 to down-modulate *Src* activity and impact on cell proliferation and invasion. The strong impairment of motility upon U94 expression was concomitant to a reduced phosphorylation of β-catenin and to down-modulation of STAT3, Cortactin and Arp2/3. Moreover, U94^+^ cells cultured in 3D fluid-dynamic condition showed disappearance of characteristic tumor cell atypias that included a decreased appearance of spindle shaped cells, indicating a switch from a highly aggressive phenotype to a less aggressive one. β-catenin re-localization at the cell surface of U94^+^ cancer cells together with a generally reduced cytoplasmic expression of vimentin was highly suggestive of MET. Indeed, molecular analysis confirmed down-modulation of the mesenchymal marker N-cadherin, accompanied by down-regulation of Twist and Snail1, both linked to EMT [33–35]. On the other hand, these cells did not show any re-expression of E-cadherin, a molecule essential for intercellular adhesion junctions and intimately connected to a cell degree of epitheliality in both morphology, migratory and invasive ability. E-cadherin expression is silenced by DNA hypermethylation [36] but it is also known that this epigenetic phenomenon can be reversed by microenvironmental factors [37]. Thus, our data indicate the incapability of U94 to trigger E-cadherin promoter demethylation and attest for the ability of viral protein to sustain a partial MET. One possible explanation for this phenomenon may reside in the lack of FAK and ERK down-modulation. In fact, both signaling pathways are known to be strongly involved in EMT [38, 39]. Introduction of tumor cells into a secondary organ environment led to the passive loss of methylation of the E-cadherin promoter and re-expression of this cell-cell adhesion molecule [37]. In fact, recent findings suggest that only non-EMT cells that have entered in the blood stream are able to reestablish colonies in the secondary sites, in light of cooperation of tumor cells with the microenvironment to become non-invasive elements with acquired cell-cell adhesion and complete the process of metastasis [40]. Surprisingly, U94^+^ cells showed a strongly decreased capability of forming metastases in lung when they were directly inoculated into the bloodstream as compared to control cells. The initial arrest and adhesion of cancer cells to the vascular endothelium, as well as vascular permeability, are essential steps preceding cancer cell extravasation from the blood stream. In this respect, it is worth noting that *Src* plays a part in the endothelial permeability by promoting pathologic inflammatory processes [41, 42]. The decreased capability of U94^+^ cells to generate lung metastases may be at least in part due to *Src* inhibition. However, our finding is also suggestive of a role for U94 in impairing tumor cell survival and/or growth during the metastatic process. In fact, U94 negatively impacts on Twist transcription, whose inhibition is known to reduce cancer cell proliferation and increase cell death [35]. *In vivo* experiments highlighted the potent and prolonged antitumor activity of U94. Despite the relatively short time expression of U94 transcripts in amplicon transduced cells (2-4 days), a long-term control of tumor growth and invasion is maintained, with significantly smaller tumor masses and in the absence of adipose tissue infiltration at the end of the study (23 days), compared to tumors generated by control cells. In particular, spindle cells with mesenchymal-like features were abundant in control tumors whereas they disappeared in the U94-conditioned ones. Moreover, angiogenesis was pronounced in control tumors but limited to few tiny vessels in those conditioned by U94. In this respect, it is worth noting that U94^+^ tumor cell conditioned medium was impairing the capability of ECs to migrate and promote angiogenesis *in vitro*. This finding adds more emphasis to our previous data showing that U94 is capable of rendering ECs insensitive to different pro-angiogenic stimuli, included VEGF [8]. The ability of U94 to condition the microenvironment and impact on EC angiogenetic activity both *in vitro* and in long-term *in vivo* experiments, is completely new and calls for further investigation aimed to identify and characterize mechanisms for such a relevant anticancer activity. U94-mediated impairment of cancer progression through a “two compartments” activity may provide anticancer therapeutic benefits not only in terms of oncosuppressive effects on tumor cells but also by inhibiting the neovascularization process, thus hampering the tumor parenchymal/stromal cross-talk.

In conclusion, data presented in this study demonstrate that an HSV-1-based amplicon vector serves as a gene delivery system for U94. The ability of U94 to block cancer cell growth and metastasis by promoting a partial MET, together with its ability to impair vasculogenesis, highlights the complex antitumor activity of a virally-encoded protein, thus opening the way to a new promising field of research. Interestingly, U94 is homologous to Rep78, a non-structural protein from the human parvovirus adeno-associated virus type 2 [43] that displays antiproliferative effects on tumor cells [44]. This evidence indicates that U94 and Rep78 constitute a first group of viral proteins that have evolved mechanisms to control cell proliferation and could therefore be harnessed for cancer therapy. Overall, our findings open the way to study viruses that normally live in and among us for genetic blueprints that enable them to make molecules that act like drugs and might serve as the basis for new human anticancer therapeutics.

## MATERIALS AND METHODS

### Cell cultures

Human breast cancer cells (MDA-MB 231) and human cervical cancer cells (HeLa) were obtained from the American Type Culture Collection and grown as described. Human umbilical vein endothelial cells (HUVECs) and african green monkey kidney cells (Vero 2-2) were cultured as previously described [6, 45].

### Construction, production and titration of U94-expressing herpes simplex type -1 (HSV-1) amplicon vectors

U94 (originated from HHV-6 type B) was amplified with the following primers: the forward primer, 5'-CTTTGTCGACACCATGTTTTCCATAATAAATC-3'; and the reverse primer, 5'-CTTTGTCGACTTATAAAATTTTCGGAACCGTG-3' using plasmid U94 pSR2PH vector as template (8). The PCR product was inserted into pHSV_S_, which contains an internal ribosomal entry site (IRES) of poliovirus [45] and supports the simultaneous expression of U94 and EGFP. Helper virus-free HSV-1 amplicon vector stocks were prepared as previously described [46]. The HSV-1 genome was provided in trans by a bacterial artificial chromosome (BAC) containing the HSV-1 genome with deletions in the DNA cleavage/packaging signals and the essential ICP27 gene (fHSVΔpacΔICP27). Vero 2-2 cells were co-transfected with amplicon plasmid DNA, the fHSVΔpacΔICP27 BAC DNA, and plasmid pEBHICP27 (which provides the HSV-1 ICP27 gene in trans), using Lipofectamine LTX and Plus Reagent (Life Technologies). After 72 h, cells were lysed and debris were removed by centrifugation. Vero 2-2 cells were infected with the amplicon stocks and, after 24 h, green fluorescent cells were counted using an inverted fluorescence. The titers were determined as transducing units (TU)/ml and ranged between 2 × 10^6^ and 8 × 10^6^ TU/ml.

### Cell infection

Infection of tumor cells was performed by incubating cells for 3 h in serum-free medium containing or not U94 and/or EGFP-expressing amplicons at different multiplicity of infection (MOI). Cells were then washed and incubated for 24 h in complete medium. Infection efficiency was assessed by flow cytometry.

### Real-time and RT-PCR analysis

Total RNA was extracted from cells with RNeasy Plus Mini Kit (QIAGEN) and reverse transcribed. The following primers were used to perform PCR: U94:the forward primer, 5'-TCTCTAACGTGTCCGTGCC-3'; and the reverse primer, 5'*-* CATCGCATACGTCTCCCAG-3'; human β-actin: the forward primer, for 5'-GGCACC CAGCACAATGAAG-3', and the reverse primer 5'-GCT GATCCACATCTGCTGG-3'. Real-time PCR was performed using the following primers: human TWIST: the forward primer, 5'-CAGACGCAGCGGGTCATG-3'; and the reverse primer, 5'-AGGGCAGCGTGGGGATGA-3'; human SNAIL1: the forward primer, 5'-TGACCTGTC TGCAAATGCTC-3'; and the reverse primer, 5'-CAGA CCCTGGTTGCTTCAA-3'; human MMP2: the forward primer, 5'-GTATGGCTTCTGCCCTGAGA-3'; and the reverse primer, 5'-CACACCACATCTTTCCGTCA-3'; human N-cadherin: the forward primer, 5'-CAACTT GCCAGAAAACTCCAGG-3'; and the reverse primer, 5'-ATGAAACCGGGCTATCTGCTC-3'; human GAPDH: the forward primer, 5'-GAAGGTCGGAGTCAAC GGATT-3'; and the reverse primer, 5'-TGACGG TGCCATGGAATTTG-3'.

### Cell viability assay and flow cytometric assay of DNA content

Cell viability was evaluated with the Cell Titer–Glo^®^Luminescent Cell Viability Assay (Promega). Twenty-four h p.i., EGFP^+^ and U94^+^ cells, as well as NT cells, were seeded in 96-well plates at a density of 5 × 10^3^ cells/well. The CellTiter–Glo reagent was then added to each well at different time points and lysis reaction was carried out for 10 min on an orbital shaker. ATP luminescence intensity was measured using the GloMax® 20/20 Luminometer. Twenty-four h p.i., EGFP^+^, U94^+^ and NT cells were also seeded in 24-well plates at a density of 1 × 10^5^ cells/well and passaged 1:2 when they were grown to approximately 80% confluence. At the indicated times, cells were trypsinized, inactivated with the addition of serum and counted using trypan blue exclusion. For performing flow cytometry, harvested cells were washed in PBS, resuspended in PBS containing 0.5% EDTA (SigmaAldrich) and fixed in 99% ethanol for 12 h at 4°C. Then cells were stained for 3 h at 4°C with PBS containing 2% FBS, 12.5 μg/ml DNase-free RNase A (SigmaAldrich) and 40 μg/ml of propidium iodide (SigmaAldrich). The percentage of G_1_, S, and G_2_/M phases of the cell cycle was measured using the MACSQuant® Analyzer (Miltenyi Biotec) and analysed using the FlowJo vX.07 software (Tree Star).

### Wound healing assay

The wound healing assay was performed as previously described [47]. Briefly, cells were cultured into 24-well plates until confluence. Twenty-four h p.i., the monolayer was scratched using a 200 μl pipette tip and cultured in complete medium. In some experiments, confluent HUVEC monolayers were scratched and cultured in the presence of conditioned medium from infected or not infected tumor cells. Cell migration was evaluated at different time points.

### Cell motility assay

Cell motility assay was performed as previously described [8]. Briefly, cells were seeded on the bottom of the flasks at a concentration of 10^5^ cells/flask and allowed to adhere by overnight incubation at 37°C. Twenty-four h p.i., flasks were positioned at an ~ 20°C angle. At day 8, migrated cells were stained with Diff-quick (Medion-Diagnostics). Cell motility rates were analyzed by measuring the distance from the edge of the flask to the leading edge of the cells.

### Invasion assay

Cell invasion assay was carried out by Matrigel-coated transwell system. Polycarbonate transwell filters (8 μm pore size, Corning) were coated with 50 μg of basement membrane extract (BME; 10 mg/ml; Cultrex) diluted in a total volume of 150 μl of serum-free medium. Then the transwells were placed in a 24 well/plate. Twenty-four h p.i., EGFP^+^ and U94^+^ cells, as well as not treated (NT) cells were serum starved for 24 h, trypsinized, resuspended in 150 μl of serum-free medium and seeded into the coated filter at a concentration of 10^4^ cells/well. Six hundred μl of complete medium were added into the lower chamber to take advantage of FBS as chemoattractant factor. The plate was incubated at 37°C and allowed to migrate through the matrigel-coated filter. After 48 h of incubation, cells that had crossed the filter were fixed, stained with Diff-Quick and counted.

### Soft agar anchorage-independent growth assay

Soft agar assay was performed as previously described [48]. Twenty-four h p.i., EGFP^+^ and U94^+^ cells, as well as not treated (NT) cells (500/well) were plated in 2 ml of 0.5% agarose, 5% charcoal-stripped FBS in DMEM, with a 0.7% agarose base in six-well plates. One day after plating, complete medium was added to the top of the layer and replaced every four days. After 10 days, 200 μl of 3-[4, 5-Dimethylthiazol-2-y1]-2, 5-diphenyltetrazolium bromide (MTT, Sigma) were added to each well and allowed to incubate for 4 h at 37°C. Colonies >50 mm diameter were counted.

### Western blot analysis

Cells (2×10^6^) were infected or not with different amplicons, cultured for 24 h and then processed as previously described [49]. The blots were incubated overnight at 4°C with mAbs to *Src* pTyr418 and Src (Invitrogen), β-catenin pTyr654 and β-catenin (Abcam), FAK pTyr397 and FAK (Invitrogen), STAT3 pTyr705 and STAT3 (Cell Signaling), Cortactin pTyr421 and Cortactin (Cell Signaling), ARP3 and GAPDH (Cell Signaling), Akt pSer473 and Akt (Cell Signaling), or ERK pThr202 and ERK (Santa Cruz Biotechnology). The antigen–antibody complex was detected using peroxidase-conjugated goat anti-mouse or anti-rabbit IgG (Thermo Scientific) and revealed using the enhanced chemiluminescence (ECL) system (Santa Cruz Biotechnology).

### 3D cell culture

Twenty-four h p.i., EGFP^+^ and U94^+^ cells, as well as not treated (NT) cells, were trypsinized and seeded at the density 1×10^6^ cells/ml in the culture chamber of the Rotary Cell Culture System (RCCS™) allowing the culture in 3D dynamic microenvironment. Three D cultures were performed for 48 h in the RCCS bioreactor.

### *In vivo* studies

Animal experiments were approved by our local animal ethics committee and were executed in accordance with national and international laws and policies (EEC Council Directive 86/609, OJ L 358, 12 December 1987). Twenty-four h p.i., EGFP^+^ and U94^+^, as well as not treated (NT), MDA-MB 231 (5×10^6^) or HeLa (3×10^6^) cells were injected into the dorsolateral flank of 7 week-old NOD/SCID female mice. Tumors were measured in two dimensions and tumor volume was calculated according to the formula V=(D x d^2^)/2, where D and d are the major and minor perpendicular tumor diameters, respectively. At the end of the experimental procedure tumors were harvested, weighted, photographed and paraffin-embedded for IHC.

For experimental metastases, NT, EGFP^+^ or U94^+^ cells (1×10^6^) were suspended in 100 μl of PBS and injected into the tail vein of 7 week-old NOD/SCID female mice. After 5 weeks lungs were harvested, formalin-fixed and the number of metastases were counted under a dissecting microscope.

### IHC

After 3D culture and tumor growth in mice, cells and biopsies were harvested and processed as previously described [11]. Cell pellets and biopsies were fixed in 10% buffered formalin and embedded in paraffin. Five μm thick sections were prepared and stained with H&E for morphological analysis. Sections for IHC were transferred to glass slides coated with poly-lisine, deparaffinized in 100% xylene, and rehydrated in graded ethanol. After heat-induced antigen retrieval, endogenous peroxidase activity was inhibited with 3% hydrogen peroxide for 15 s at room temperature, while aspecific epitope binding was blocked by incubation for 20 min with 20% human serum. Each section was then incubated for 30 min with monoclonal antibody to β-catenin (diluition: 1:120; Cell Marque), vimentin (diluition 1:150; Leica Biosystems), E-cadherin (diluition 1:50; Invitrogen) or with rabbit polyclonal antibody to mouse CD31 (dilution 1:50; Abcam). A biotin free polymeric-horseradish peroxidase-linker antibody conjugate system (Bond Polymer Define Detection; Leica Biosystems) was used on the Bond Max automated immunostainer (Leica). 3-3' Diaminobenzidine Tetrahydrocloride (DAB) was used as substrate chromogen and Haematoxylin for nuclear counterstaining. Appropriate negative and positive control slides were run in parallel.

### Cell co-cultivation and *in vitro* tube formation assay

Co-culture between MDA-MB 231 and HUVEC cells was performed in absence of direct cell contact by using transwell inserts (polycarbonate filters, coated with collagene, 0.4 μm pore size, Corning). Briefly, 24 h p.i., EGFP^+^ and U94^+^ as well as not treated (NT) MDA-MB 231 cells were seeded directly in the transwell bottom well and HUVECs on the collagen-coated insert. After 24 h, cells in the upper well were trypsinized and used to perform the tube formation assay [50].

### Statistical analysis

Data obtained from multiple independent experiments are expressed as the mean ± SD. Data were analyzed for statistical significance using the 1-way ANOVA, and Bonferroni post-test was used to compare data. Student's t test for unpaired data (2-tailed) was used to test the probability of significant differences between two groups of samples. Tumor volume data were statistically analyzed with a 2-way analysis of variance, and individual group comparisons were evaluated by the Bonferroni correction. Differences were considered significant when *P* < 0.05. Statistical tests were performed using GraphPad Prism 5 software.
